# The Role of Data Type and Recipient in Individuals’ Perspectives on Sharing Passively Collected Smartphone Data for Mental Health: Cross-Sectional Questionnaire Study

**DOI:** 10.2196/12578

**Published:** 2019-04-05

**Authors:** Jennifer Nicholas, Katie Shilton, Stephen M Schueller, Elizabeth L Gray, Mary J Kwasny, David C Mohr

**Affiliations:** 1 Center for Behavioral Intervention Technologies Department of Preventive Medicine, Feinberg School of Medicine Northwestern University Chicago, IL United States; 2 College of Information Studies University of Maryland College Park, MD United States; 3 Department of Psychological Science University of California - Irvine Irvine, CA United States; 4 Biostatistics Collaboration Center Department of Preventive Medicine, Feinberg School of Medicine Northwestern University Chicago, IL United States

**Keywords:** mHealth, privacy, personal sensing, digital mental health, depression, anxiety, mobile phone

## Abstract

**Background:**

The growing field of personal sensing harnesses sensor data collected from individuals’ smartphones to understand their behaviors and experiences. Such data could be a powerful tool within mental health care. However, it is important to note that the nature of these data differs from the information usually available to, or discussed with, health care professionals. To design digital mental health tools that are acceptable to users, understanding how personal sensing data can be used and shared is critical.

**Objective:**

This study aimed to investigate individuals’ perspectives about sharing different types of sensor data beyond the research context, specifically with doctors, electronic health record (EHR) systems, and family members.

**Methods:**

A questionnaire assessed participants’ comfort with sharing six types of sensed data: physical activity, mood, sleep, communication logs, location, and social activity. Participants were asked about their comfort with sharing these data with three different recipients: doctors, EHR systems, and family members. A series of principal component analyses (one for each data recipient) was performed to identify clusters of sensor data types according to participants’ comfort with sharing them. Relationships between recipients and sensor clusters were then explored using generalized estimating equation logistic regression models.

**Results:**

A total of 211 participants completed the questionnaire. The majority were female (171/211, 81.0%), and the mean age was 38 years (SD 10.32). Principal component analyses consistently identified two clusters of sensed data across the three data recipients: “health information,” including sleep, mood, and physical activity, and “personal data,” including communication logs, location, and social activity. Overall, participants were significantly more comfortable sharing any type of sensed data with their doctor than with the EHR system or family members (*P*<.001) and more comfortable sharing “health information” than “personal data” (*P*<.001). Participant characteristics such as age or presence of depression or anxiety did not influence participants’ comfort with sharing sensed data.

**Conclusions:**

The comfort level in sharing sensed data was dependent on both data type and recipient, but not individual characteristics. Given the identified differences in comfort with sensed data sharing, contextual factors of data type and recipient appear to be critically important as we design systems that harness sensor data for mental health treatment and support.

## Introduction

Personal sensing, also referred to as context sensing and digital phenotyping [1], is the acquisition and use of data from networked sensors (as in a smartphone) for the detection of behaviors, psychological states, and environmental conditions [2]. Personal sensing shows great promise within mental health research [3]. Sensed data have already been used in a number of mental health conditions including schizophrenia [4], bipolar disorder [5], social anxiety [6], and depression [7]. For example, in schizophrenia, changes in mobility and social behavior, measured using global positioning system (GPS) and communication log data, were found to proceed clinical relapse [4]. Mobility and location data, measured using information extracted from GPS data [8,9] or the number of cell tower connections [5], have also been reported to identify and predict episodes of depression in bipolar disorder and are associated with severity of unipolar depression symptoms [9-11]. Sleep duration can also be successfully inferred using sensed data acquired from smartphones [12-14] and is related to depression severity [15]. Finally, data on subjectively reported mood, collected via ecological momentary assessments, that often accompany sensed data have demonstrated validity, correlating highly with clinician-assessed mood scales [5]. However, as demonstrations of the potential of sensed data to support mental health care and behavior change increase, questions arise regarding the acceptability of collecting different types of sensed data and the people who have access to that information.

Attitudes about privacy related to digitally collected data are theorized to rely on two major variables: contextual factors and individual characteristics. Research indicates that contextual factors may be the primary influence on people’s reasoning about privacy [16,17]. A robust framework—privacy as contextual integrity—defines the contextual factors that influence people’s privacy judgements and willingness to share data, such as data type and sensitivity, data use, transmission principles and constraints (eg, confidentiality or anonymity), and data recipient [18,19]. In mental health research, the *sensitivity* of data is high [20]. The *types* of data collected for mental health are broad, ranging from mood, communication logs, and social activity to GPS data. Finally, within the mental health field, potential data *recipients* beyond researchers include doctors, electronic health record (EHR) systems, and family members. Importantly, the digital data privacy literature emphasizes the need for individuals to understand (and preferably control) who has access to their personal data [16,21,22].

Despite the importance of privacy considerations in personal sensing, few studies have explored how contextual factors such as data type or recipient influence the acceptability and appropriate use of smartphone sensor data in the treatment and management of mental health. The closest example is Klasnja and colleagues’ [23] study of sensed data related to physical activity. Participants’ concerns about personal sensing varied depending on the type of sensor data collected. Accelerometer data were not considered sensitive, so their daily recording and storage were not of concern. However, perspectives on the collection and storage of GPS data were mixed, and raw audio data were considered very sensitive, with most participants indicating that they would not allow continuous recording. Although these results lend support to the privacy as contextual integrity framework and provide insight into privacy perspectives in personal sensing, many contextual factors differ between sensed data collected for physical activity and mental health purposes, which potentially impact the extensibility of the findings to the mental health context.

In addition to contextual factors, individual characteristics of users may influence attitudes towards privacy. Although, to our knowledge, no research has assessed differences in perspectives of sensed data privacy between people living with and those not living with a mental health condition, research indicates that data privacy and confidentiality are among the primary concerns of individuals with a mental health condition when considering the use of apps to support their mental health [24,25]. Research also suggests that older individuals may have more concerns about the collection and sharing of sensed data than younger people [26]. In a study of app privacy permissions, including access to sensors, participants characterized as “unconcerned” by permissions (those who had a high comfort level with sharing sensitive information across numerous settings) were more likely to be younger. Characteristics of the individual should therefore be considered in conjunction with context when exploring perspectives regarding sensed data sharing in mental health.

As advances in personal sensing aim to integrate the passive identification of behavioral indicators of common mental health disorders such as depression and anxiety with existing mental health services, it is critical to understand how context and individual characteristics influence individuals’ perspectives regarding the use and sharing of sensed data. Understanding such perspectives is vital to guiding the design and successful implementation of digital mental health systems. The aims of this study were (1) to investigate the acceptability of sharing sensed mental health data beyond researchers, specifically with doctors, EHR systems, and family members; (2) to determine the acceptability of use of different types of sensed data beyond the research context by doctors, EHR systems, and family members; and (3) to explore the impact of age and presence of anxiety or depression on the acceptability of sharing data.

## Methods

### Participants

Data were collected from a convenience sample of individuals participating in a 6-week personal sensing study that required them to download an app that collected mobile sensor data including activity, light, GPS location, and communication logs, and to complete daily questionnaires regarding sleep and wake times [12]. In that study, participants were eligible if they were aged 18 years or above, were able to read and understand English, owned an Android smartphone, and had access to Wi-Fi connectivity for at least a 3-hour period each day. Participants were excluded if they were diagnosed with any psychotic disorder or screened positive for a substance use disorder (Alcohol Use Disorder Identification Test [27] score ≥16 or Drug Abuse Screening Test-10 [28] score ≥6), suicidal ideation (Patient Health Questionnaire - 9 item [PHQ-9] [29], item 9 rating ≥1 or Beck Depression Inventory-II [30], item 9 rating ≥2), or bipolar disorder (mood disorder questionnaire [31] question 1 rating ≥7, endorsed question 2, and responded 2 or 3 for question 3). Individuals who shared their smartphone with others were also excluded.

Based on the results of the PHQ-9 and Generalized Anxiety Disorder - 7 item (GAD-7) [32] screening questionnaires, participants were selected to create roughly four equal groups across depression and anxiety symptoms: nondepressed or anxious (PHQ-9 score<10; GAD-7 score<10), depressed (PHQ-9 score ≥ 10; GAD-7 score <10), anxious (PHQ-9 score <10; GAD-7 score ≥10), and depressed and anxious (PHQ-9 score ≥ 10; GAD-7 score ≥10).

The study was approved by the Northwestern University Institutional Review Board. Participant responses from only the screening and baseline questionnaire were considered in this study. Within the larger study, participants were compensated for their participation. Compensation depended on both the length of their participation in the study and the number of daily questionnaires answered, and ranged between US $25 and US $270.40.

### Measures

Demographic information (for example, age and sex) and data on the presence of depression or anxiety, determined using the PHQ-9 and GAD-7, were collected by self-report.

The acceptability of sharing sensed data was measured through a series of questions with regard to three potential recipients: the participant’s doctor, representing a known individual in the health care system; the participant’s EHR system, a generic destination in the health care system that would broaden access to potentially unknown people; and the participant’s family members. Response options were recorded on an ordinal scale:

0 - I would not use any app that gave these data to my doctor/electronic health record/family member;

1 - I’d be uncomfortable but would consider using an app that did this;

2 - It wouldn’t matter to me;

3 - I’d like an app that gave these data to my doctor/electronic health record/family members.

Participants rated their comfort with sharing five classes of sensed activities or states (physical activity, sleep, mood, social activity, and location [places visited and patterns of movement]) and one raw sensed data type (communication logs [number of calls made or texts sent]), with each of the three recipients.

### Data Analysis

Given the ordinal (0, 1, 2, and 3) nature of the responses on the acceptability scale and the relatively limited sample size, it was not appropriate to treat these data as continuous. Although these outcomes are appropriate for ordinal logistic regression, we did not assume proportional odds for our models, and hence, survey responses were dichotomized to indicate whether the participant was comfortable sharing their data. Responses 0 and 1 (*I would not use any app that gave these data to my doctor/electronic health record/ family member* and *I’d be uncomfortable but would consider using an app that did this*, respectively) were coded as not comfortable, and responses 2 and 3 (*It wouldn’t matter to me* and *I’d like an app that gave these data to my doctor/electronic health record/ family members*, respectively) were coded as comfortable.

First, we performed a series of three principal component analyses, one for each data recipient, to identify clusters of sensor data types according to participants’ comfort with sharing them. Second, we determined relationships between recipients and the types of sensor data using generalized estimating equation logistic regression models of each participant’s 18 dichotomized responses (3 types of recipients × 6 types of sensor data, which were clustered into two groups based on the principal component analysis results). Third, we explored the influence of age and mental health on participants’ comfort with sharing sensed data by adding age, depression (defined as PHQ-9 score ≥ 10 and GAD-7 score <10), and anxiety (defined as PHQ-9 score <10 and GAD-7 score ≥10) as covariates in the model. Models were assessed using analysis of variance and Wald tests. All analyses were performed using R (v3.4.3; R Foundation for Statistical Computing, Vienna, Austria) with a type I error rate of 0.05.

## Results

### Participants

A total of 211 eligible participants were enrolled and completed the privacy survey. The majority were female (171/211; 81.0%), and the mean (SD) age was 38 (10.32) years, ranging from 18 to 66 years. A total of 83% (176/211) of participants identified as Caucasian; 13.3% (28/211), as African American; and 9.0% (19/211), as Hispanic or Latino. Further details of the sample are shown in Table 1.

**Table 1:** Participant characteristics.

Characteristic		Statistics
**Gender, n (%)**		
	Male	36 (17.1)
	Female	171 (81.0)
	Another	3 (1.4)
Age (years), mean (SD)		38.09 (10.32)
**Race and ethnicity^a^, n (%)**		
	Black or African American	28 (13.3)
	American Indian or Alaska native	6 (2.8)
	Asian	10 (4.7)
	White	176 (83.4)
	Hispanic or Latino	19 (9.0)
**Highest level of education, n (%)**		
	Some high school	4 (1.9)
	Completed high school	25 (11.8)
	Some college	77 (36.5)
	Completed associate’s or bachelor’s degree	77 (36.5)
	Master’s degree	23 (10.9)
	Doctoral degree or professional doctorate	5 (2.4)
**Employment status, n (%)**		
	Employed	130 (61.6)
	Unemployed	44 (20.9)
	Disability	17 (8.1)
	Retired	4 (1.9)
	Other	16 (7.6)
**Mental health status, n (%)**		
	Healthy (PHQ-9^b^ score<10 and GAD-7^c^ score<10)	59 (28.0)
	Depressed (PHQ-9 score≥10 and GAD-7 score<10)	55 (26.1)
	Anxious (PHQ-9 score<10 and GAD-7 score≥10)	44 (20.9)
	Depressed and anxious (PHQ-9 score≥10 and GAD-7 score≥10)	53 (25.1)

^a^Race and ethnicity categories are not mutually exclusive.

^b^PHQ-9: Patient Health Questionnaire - 9 item.

^c^GAD-7: Generalized Anxiety Disorder - 7 item.

### Sensor Data Type

Principal component analyses were performed on participant responses to their comfort level with sharing the six types of sensor data with each of the three data recipients. Although sensor clusters were largely consistent across data recipients, the relative strengths of each variable across recipients differed. The two clusters extracted across each of the recipients could best be described as “health information,” including sleep, mood, and physical activity, and “personal data,” including communication logs, location, and social activity.

**Figure 1:**
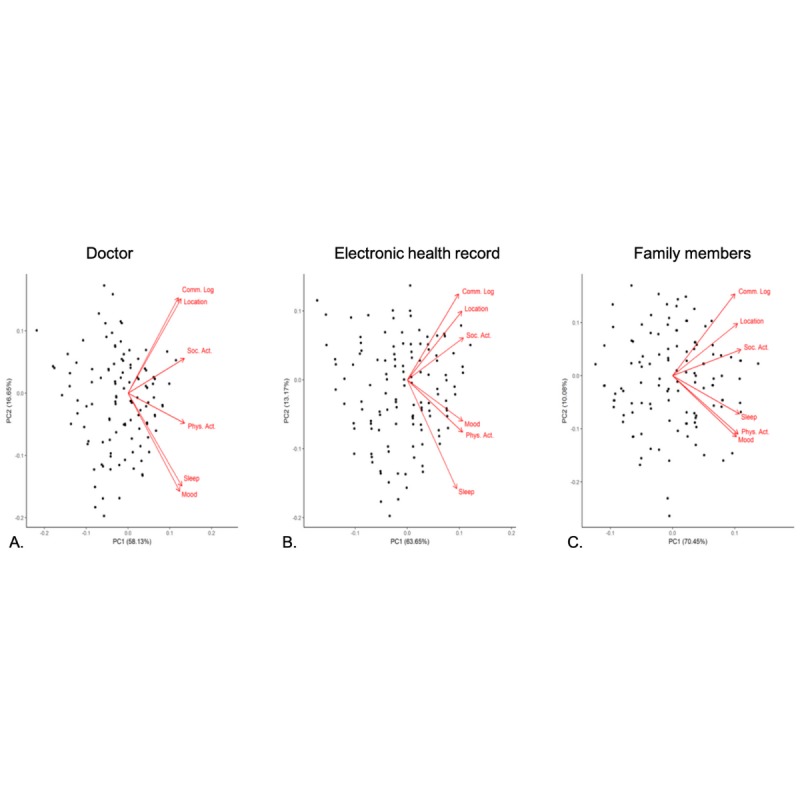
Principal component analysis of participant comfort with sharing data from six different sensors with their doctor, electronic health record, and family members. Comm Log; communications log; PC: principal component; Phys Act: physical activity; Soc Act: social activity.

For doctor recipients, the two principle components explained 74.8% of the variability in the responses (Figure 1). Participants were most comfortable sharing health information with doctors and least comfortable sharing personal data, particularly communication logs and location. The same two components for the EHR system explained 76.8% of the variability in the responses (Figure 1). Participants were least comfortable sharing personal data with their EHR system. For family members, the two components explained 80.5% of the responses (Figure 1); however, the two groups were less distinct in terms of comfort with sharing personal data with family members than with doctors or EHR systems.

### Sensor Data Sharing

Comfort level in sharing the different types of sensor data with each of the three recipient groups is displayed in Table 2. On the whole, participants indicated they were comfortable sharing their sensor data apart from communication logs and location data, and the comfort levels were higher when such data were shared with their doctor than with their family members.

**Table 2:** Participants’ comfort level sharing various sensor data with different recipients. The values indicate participants who were comfortable with sharing data.

Sensor data type	Doctor, n (%)	Electronic health record, n (%)	Family members, n (%)
Sleep	188 (89.1)	183 (86.7)	135 (64.0)
Mood	173 (82.0)	151 (71.6)	108 (51.2)
Physical activity	162 (76.8)	152 (72.0)	109 (51.7)
Communication logs	135 (64.0)	110 (52.1)	115 (54.5)
Location	122 (57.8)	105 (49.8)	97 (46.0)
Social activity	154 (73.0)	129 (61.1)	116 (55.0)

Generalized estimating equation logistic regression models were fit on the binary outcome of participants’ comfort with sharing sensor data, with the type of sensor data, the recipient of sensor data, presence of depression, presence of anxiety, and age (modeled continuously) used as covariates. The interaction of depression and anxiety was considered, but was not significant (*P*=.25). As responses on the sensor data type were highly correlated, the type was grouped according to the results of the principal component analyses mentioned above, with sleep, mood, and physical activity combined as “health information” and communication logs, location, and social activity grouped as “personal data.” Data recipient was a significant predictor of a participant’s comfort with sharing data (*P*<.001; Table 3). Individuals were significantly more comfortable sharing their sensed data with their doctor than with family members (*P*<.001) or their EHR system (*P*<.001), regardless of the data type. As suggested by the principal component analyses, overall, participants were significantly more likely to be comfortable sharing “health information” than “personal data” (*P*<.001). The model results also indicated a significant interaction between data type and recipient (*P*<.001). Comfort with sharing data was more strongly associated with data recipient for health information than for personal data, such that the difference in comfort with sharing data between doctors and family members was greater for health information than for personal data. We failed to detect any difference in comfort with sharing data with regard to depression (*P*=.12), anxiety (*P*=.14), or age (*P*=.67) in our study.

**Table 3:** Model summary of participants’ comfort with sharing “health information” (sleep, mood, and physical activity) and “personal data” (communication logs, location data, and social activity) with their doctor, the electronic health record system, or family members.

Covariate	Estimate	SE	Wald statistic	Pr(>|W|)
Intercept	0.745	0.468	2.529	.112
Age	–0.002	0.011	0.041	.840
Anxiety	0.309	0.211	2.158	.142
Depression	–0.333	0.214	2.416	.120
Health information^a^	0.953	0.126	57.621	<.001^b^
Personal data: Recipient - EHR^c,d^	–0.451	0.074	36.722	<.001^b^
Personal data: Recipient - family^e^	–0.554	0.108	26.460	<.001^b^
Health information: Recipient - EHR^c^	0.086	0.103	0.690	.406
Health information: Recipient - family^e^	–0.801	0.141	32.453	<.001^b^

^a^Health information versus personal data.

^b^These values are significant.

^c^EHR versus doctor as recipient.

^d^EHR: electronic health record.

^e^Family versus doctor as recipient.

## Discussion

### Principal Findings

This study explored the attitudes of participants toward sharing of personal data gathered from smartphone sensors with three potential data recipients in the context of mental health care involving the use of digital interventions. We found that the level of comfort with sharing sensed data was dependent on both data *type* and *recipient*, but not individual characteristics. This result is in accordance with the contextual integrity framework [18,33], which states that privacy expectations are influenced by contextual factors such as data type and sensitivity, the relationship between the individual and the data recipient, and context-specific information norms [33]. We found that participants had similar levels of comfort with sharing sleep, mood, and physical activity data (“health information”) and were more comfortable sharing them than communication logs, location, and social activity data (“personal data”). Moreover, participants were significantly more comfortable sharing sensed data with their doctor than with the EHR system or family members.

This difference between comfort in sharing data with doctors and that in sharing data with the EHR system particularly emphasizes the nuanced role that the data recipient plays in privacy concerns. The difference is possibly dependent on the existing relationship between the individual and the data recipient, and the important role of trust in privacy [33]. Participants may attribute more trust to a specific person with whom a relationship discussing health has been established (ie, their doctor), rather than the health system more generally, represented by the EHR system. Indeed, previous research has shown that when a trusting relationship is not established, participants resist sharing sensed physical activity data with health providers [34]. In our study, the fact that participants did not always extend trust and willingness to share their data with the EHR system may indicate that they have concerns about who can access their data.

The role of the recipient in comfort with data sharing, therefore, has important implications for the use of apps that acquire and transmit sensed information related to mental health, especially given the variety of people who are often involved in mental health treatment and management. Providers as well as technological system designers must be aware that although individuals may be comfortable sharing their sensed data with their doctor, they may not be comfortable sharing it more widely, even with people who are already involved in their mental health management such as other health professionals, via the EHR system, or family members. As apps are integrated into clinical care, upfront and ongoing conversations regarding the distribution of sensed data will become increasingly critical, as will provider education about sensed data and the ability for providers and individuals to manage sharing options.

In line with contextual integrity, participants’ comfort with sharing sensed data significantly differed by data type. The way in which these different data types fit within existing information norms in the doctor-patient context could possibly explain the observed differences. Sleep, mood, and physical activity data types may closely align with doctor-patient information norms, as they are often discussed with providers. On the other hand, the data types that participants were least comfortable in sharing—communication logs, location, and social activity—are not commonly discussed with doctors. Thus, discussing these data types may violate existing norms and therefore be less willingly shared with providers.

Another explanation for the observed difference in comfort with sharing across data types is that communication logs, location, and social activity data may carry additional sensitivities. For example, just four points of GPS data can reveal the identity of up to 95% of individuals [35]; personal communications document a wider variety of behaviors, habits, and beliefs than data like sleep patterns, and sharing social activity data might put the privacy of other individuals at risk. Such differences related to data type, information norms, and the unique sensitivities associated with certain data are critical to understand and heed as we begin to create systems that harness sensor data for mental health treatment and support.

Comfort with data sharing could not be predicted based on the individual characteristics of age and presence of depression or anxiety, supporting an important tenet of contextual integrity: Contextual factors more strongly influence individuals’ privacy preferences than individual characteristics [36]. The lack of differences in comfort with sharing sensed data between individuals with depression or anxiety and those without a mental health condition found in our study is interesting and important, given the stigmatized nature of these common mental health conditions [37] and the risks associated with sharing. However, further research should aim to elucidate whether any differences in the nature of concerns regarding data privacy exist between people with common mental health disorders and those without such disorders, in order to mitigate any specific concerns of this population.

Irrespective of people’s mental health status, ethicists have raised concerns that personal sensing projects challenge traditional research ethics’ tenets of informed consent and risk mitigation, because data collection is both unobtrusive (easily forgotten and not easily avoided) and pervasive (recording many aspects of a participants’ daily habits for long periods) [19,38], and the inferential harms of passively collected data are often poorly understood [39]. Our findings suggest that personal sensing projects should use contextual factors to guide research design and should revise participant consent processes to address these ethical concerns. For example, individuals should be afforded the opportunity to select specific allowances for data sharing based on factors such as type, purpose, recipient, and sensitivity, rather than providing a blanket consent. Further, researchers should not assume that the acceptability of using sensed data is easily generalized between different research contexts or types of data collected without first considering the comparability of the contextual norms (roles, data types, transmission principles, and uses). Beyond these considerations, data collectors must also ensure protections, such as data deletion, deidentification, and restrictions on sharing.

### Limitations

Although this study reveals important differences in the comfort level of individuals sharing sensed data based on the recipient and data type, a number of limitations should be considered when interpreting the results. First, survey participants consented to participate in a wider study that collected data from a number of smartphone sensors over a 6-week period. Therefore, the views of individuals who were deeply uncomfortable with sharing sensed data were likely not represented within the sample. This sampling bias reduces the generalizability of these findings to the general population. However, these findings do represent sensitivities of people who are open to using personal sensing apps. As a further caution to generalizability, we note that the majority of the sample comprised white, employed, and well educated people. Given that privacy is experienced by different populations in distinct ways [40], further work should examine privacy associated with mental health across a broader section of the community.

This study asked general questions about individuals’ willingness to share sensed data and did not explore richer contextual factors such as perceived benefits or risks that are often considered by individuals when making decisions about privacy [39]. Therefore, the behavior and decisions made by individuals when deciding whether to share sensed data may vary from what is outlined here. The importance of richly contextualizing information about the collection of sensed data for mental health was highlighted by a recent study [34] on veterans with posttraumatic stress disorder. Researchers found that the lack of clarity of purpose was a primary reason given by participants for not using a wearable fitness tracker to support treatment. Lack of purpose also contributed to uncertainty and increased discomfort about the collection of sensed GPS and Bluetooth data in a feasibility study of a passive data–collection app [41]. Individuals’ existing beliefs about the importance of privacy and data control have also been shown to interact with contextual factors when people make privacy decisions [36]. These beliefs were not explored in the current study. Future research that more richly contextualizes the collection and sharing of sensed data and explores existing beliefs using vignettes, semistructured interviews, or other contextualized methods would provide deeper insight into why the reported differences in comfort with sharing sensed data exist.

Finally, considering the importance of context in our study, it is worth noting that the data in this study were collected before a number of highly publicized data privacy scandals took place, most notably, that of Cambridge Analytica, in which data were misused, and that of Strava, where released data had unforeseen consequences for disclosure. These events brought to light the importance of contextual factors such as unforeseen harms and data re-identification, purpose, and recipient. Individuals’ attitudes toward passive data collection may have since changed, again highlighting the need for further contextualized research regarding the privacy of sensed data for mental health.

### Conclusions

In line with the contextual integrity framework, participants’ comfort with sharing sensed data was dependent on the type of data collected and the intended recipient of those data. Given these differences, research and treatment protocols and systems designed to use sensed data must consider differences in individuals’ comfort depending on contextual factors. These differences represent important considerations, as systems are developed to integrate sensed data into health systems and use these data to encourage behavior change and mental health management. The reported insights will help establish data sharing norms for personal sensing and manage or mitigate privacy concerns as we develop systems to collect, share, and use sensed data to support mental health treatment.

## Abbreviations

EHR: electronic health record
GAD-7: Generalized Anxiety Disorder - 7 item
GPS: global positioning system
PHQ-9: Patient Health Questionnaire - 9 item
